# 2-Hydr­oxy-3-nitro­benzamide

**DOI:** 10.1107/S1600536809022843

**Published:** 2009-06-20

**Authors:** Abdul Rauf Raza, Muhammad Danish, M. Nawaz Tahir, Bushra Nisar, Mohammad S. Iqbal

**Affiliations:** a Department of Chemistry, University of Sargodha, Sargodha, Pakistan; b Department of Physics, University of Sargodha, Sargodha, Pakistan; c Department of Chemistry, Government College University, Lahore, Pakistan

## Abstract

The asymmetric unit of title compound, C_7_H_6_N_2_O_4_, contains two mol­ecules, one of which has a disordered nitro group with an occupancy ratio of 0.517 (9):0.483 (9) for the O atoms. Both mol­ecules contain an intra­molecular O—H⋯O hydrogen bond. In the crystal, both mol­ecules form inversion dimers linked by pairs of N—H⋯O hydrogen bonds, resulting in *R*_2_^2^(8) ring motifs. The dimers are connected by further N—H⋯O links and weak C—H⋯O inter­actions, resulting in a layered motif.

## Related literature

For related structures, see: Liu & Zhu (2007 ▶); Pertlik (1990 ▶). For graph-set notation, see: Bernstein *et al.* (1995 ▶).
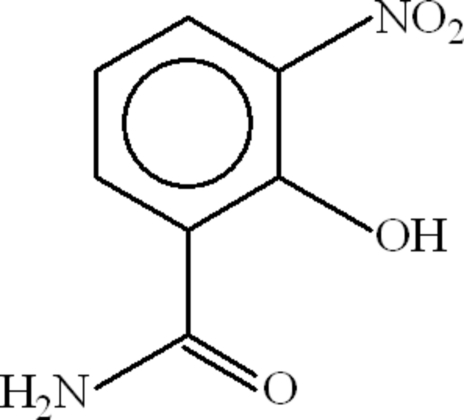


## Experimental

### 

#### Crystal data

C_7_H_6_N_2_O_4_
*M* *_r_* = 182.14
Triclinic, 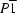

*a* = 3.8390 (2) Å
*b* = 13.0347 (8) Å
*c* = 16.0409 (9) Å
α = 98.207 (3)°
β = 95.658 (2)°
γ = 98.365 (3)°
*V* = 780.16 (8) Å^3^
*Z* = 4
Mo *K*α radiation
μ = 0.13 mm^−1^
*T* = 296 K
0.25 × 0.22 × 0.18 mm

#### Data collection

Bruker Kappa APEXII CCD diffractometer
Absorption correction: multi-scan (*SADABS*; Bruker, 2005 ▶) *T*_min_ = 0.966, *T*_max_ = 0.979
15183 measured reflections
3658 independent reflections
1932 reflections with *I* > 2σ(*I*)
*R*_int_ = 0.039

#### Refinement

*R*[*F* ^2^ > 2σ(*F* ^2^)] = 0.048
*wR*(*F* ^2^) = 0.139
*S* = 1.00
3658 reflections
287 parameters
H atoms treated by a mixture of independent and constrained refinement
Δρ_max_ = 0.19 e Å^−3^
Δρ_min_ = −0.18 e Å^−3^

### 

Data collection: *APEX2* (Bruker, 2007 ▶); cell refinement: *SAINT* (Bruker, 2007 ▶); data reduction: *SAINT*; program(s) used to solve structure: *SHELXS97* (Sheldrick, 2008 ▶); program(s) used to refine structure: *SHELXL97* (Sheldrick, 2008 ▶); molecular graphics: *ORTEP-3* (Farrugia, 1997 ▶) and *PLATON* (Spek, 2009 ▶); software used to prepare material for publication: *WinGX* (Farrugia, 1999 ▶) and *PLATON*.

## Supplementary Material

Crystal structure: contains datablocks global, I. DOI: 10.1107/S1600536809022843/hb5009sup1.cif

Structure factors: contains datablocks I. DOI: 10.1107/S1600536809022843/hb5009Isup2.hkl

Additional supplementary materials:  crystallographic information; 3D view; checkCIF report

## Figures and Tables

**Table 1:** Hydrogen-bond geometry (Å, °)

*D*—H⋯*A*	*D*—H	H⋯*A*	*D*⋯*A*	*D*—H⋯*A*
O1—H1*O*⋯O4	0.82	1.77	2.500 (2)	148
N2—H2*A*⋯O6*A*^i^	0.80 (3)	2.38 (3)	3.142 (5)	161 (3)
N2—H2*B*⋯O4^ii^	0.93 (3)	2.03 (3)	2.966 (3)	177 (2)
N4—H4*A*⋯O8^iii^	0.87 (3)	2.07 (3)	2.929 (3)	172 (2)
N4—H4*B*⋯O2^iv^	0.91 (3)	2.18 (3)	3.084 (3)	174 (2)
O5—H5*O*⋯O8	0.82	1.76	2.496 (2)	148
C6—H6⋯O6*A*^i^	0.91 (2)	2.40 (2)	3.285 (6)	163 (2)
C11—H11⋯O7*A*^v^	0.92 (2)	2.41 (2)	3.221 (6)	147 (2)
C11—H11⋯O7*A*^vi^	0.92 (2)	2.55 (2)	2.997 (6)	110.5 (17)
C13—H13⋯O2^iv^	0.92 (2)	2.39 (2)	3.289 (3)	165 (2)

Symmetry codes: (i) 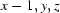; (ii) 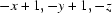; (iii) 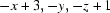; (iv) 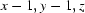; (v) 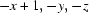; (vi) 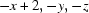.

**Fig. 1.**
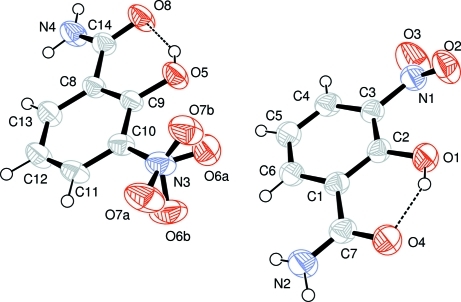
View of (I) with displacement ellipsoids drawn at the 50% probability level. H-atoms are shown by small spheres of arbitrary radius. Hydrogen bonds are symbolized by dashed lines.

**Fig. 2.**
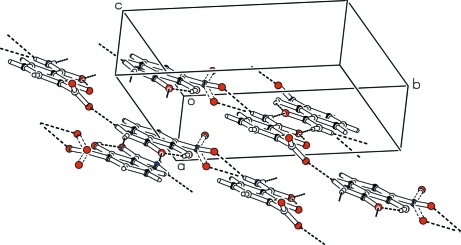
The partial packing of (I) which shows that molecules form dimers and the dimers are interlinked forming two dimensional polymeric sheets.

## supplementary crystallographic information

### Comment

The title compound (I), (Fig. 1), has been prepared as an intermediate for derivatization. The purpose of structure determination was to investigate the amount and position of nitration on the 2-hydroxybenzamide.

The crystal structures of (II) 2-Hydroxybenzamide (Pertlik, 1990) and (III) 2-Hydroxy-3,5-dinitrobenzamide monohydrate (Liu & Zhu, 2007) have been published which contain the common group of hydroxybenzamide as in (I).

The title compound consists of two molecules in the asymmetric unit. The O-atoms of nitro group in one of the molecules are disordered over two sites with occupancy ratio of 0.517 (9):0.483 (9). In both molecules the hydroxy group form intramolecular H-bonding with the O-atom of amide group, thus completing ring motifs *R*_1_^1^(6) (Bernstein *et al.*, 1995). The H-atoms of NH_2_ groups behave differently. One H-atom forms dimer, whereas the other is used in linkage of the dimers. It is intersting that both molecules form dimers among themselves through the intermolecular H-bonds of N—H···O type with ring motifs *R*_2_^2^(8). The dimers of both molecules are connected to each other through the same type of intermolecular H-bonding (Fig. 2). The molecules are stabilized in the form of two dimensional polymeric sheets due to intera as well as intermolecular H-bonding (Table 1).

### Experimental

A solution of 2-hydroxybenzamide (1.37 g, 0.01 mol) in ethylacetate (EtOAc) (25 ml) was added dropwise to a nitrating mixture of HNO_3_ (1.89 g, 0.03 mol) and H_2_SO_4_ (1.96 g, 0.02 mol) with constant stirring while the temperature was kept below 278 K. Then reaction mixture was stirred at room temperature for 4–5 h. The resulting mixture was refluxed for 1 h, cooled, neutralized with aq. NaHCO_3_ (10%) and extracted with EtOAc (3 × 25 ml). The organic layers were combined, dried over anhydrous Na_2_SO_4_, filtered and concentrated under reduced pressure to afford a reddish brown solid. The column chromatographic purification with 0, 2.5, 5 and 7.5% EtOAc in petrol (0.5 *L* each) over a silica gel packed column (25.5 cm) afforded brown prisms of (I).

### Refinement

The H-atoms were positioned geometrically, with O—H = 0.82 Å for hydroxy groups. The coordinates of all other H-atoms were refined with *U*_iso_(H) = 1.2U_eq_(carrier).

### Crystal data

**Table:**

C_7_H_6_N_2_O_4_	*Z* = 4
*M**_r_* = 182.14	*F*(000) = 376
Triclinic, *P*1	*D*_x_ = 1.551 Mg m^−^^3^
Hall symbol: -P 1	Mo *K*α radiation, λ = 0.71073 Å
*a* = 3.8390 (2) Å	Cell parameters from 3658 reflections
*b* = 13.0347 (8) Å	θ = 1.3–27.9°
*c* = 16.0409 (9) Å	µ = 0.13 mm^−^^1^
α = 98.207 (3)°	*T* = 296 K
β = 95.658 (2)°	Prism, brown
γ = 98.365 (3)°	0.25 × 0.22 × 0.18 mm
*V* = 780.16 (8) Å^3^	

### Data collection

**Table:**

Bruker Kappa APEXII CCD diffractometer	3658 independent reflections
Radiation source: fine-focus sealed tube	1932 reflections with *I* > 2σ(*I*)
graphite	*R*_int_ = 0.039
Detector resolution: 7.40 pixels mm^-1^	θ_max_ = 27.9°, θ_min_ = 1.3°
ω scans	*h* = −5→5
Absorption correction: multi-scan (*SADABS*; Bruker, 2005)	*k* = −17→16
*T*_min_ = 0.966, *T*_max_ = 0.979	*l* = −20→20
15183 measured reflections	

### Refinement

**Table:**

Refinement on *F*^2^	Primary atom site location: structure-invariant direct methods
Least-squares matrix: full	Secondary atom site location: difference Fourier map
*R*[*F*^2^ > 2σ(*F*^2^)] = 0.048	Hydrogen site location: inferred from neighbouring sites
*wR*(*F*^2^) = 0.139	H atoms treated by a mixture of independent and constrained refinement
*S* = 1.00	*w* = 1/[σ^2^(*F*_o_^2^) + (0.0563*P*)^2^ + 0.1478*P*] where *P* = (*F*_o_^2^ + 2*F*_c_^2^)/3
3658 reflections	(Δ/σ)_max_ < 0.001
287 parameters	Δρ_max_ = 0.19 e Å^−^^3^
0 restraints	Δρ_min_ = −0.18 e Å^−^^3^

### Special details

**Table:**

Geometry. Bond distances, angles *etc*. have been calculated using the rounded fractional coordinates. All su's are estimated from the variances of the (full) variance-covariance matrix. The cell e.s.d.'s are taken into account in the estimation of distances, angles and torsion angles
Refinement. Refinement of *F*^2^ against ALL reflections. The weighted *R*-factor *wR* and goodness of fit *S* are based on *F*^2^, conventional *R*-factors *R* are based on *F*, with *F* set to zero for negative *F*^2^. The threshold expression of *F*^2^ > σ(*F*^2^) is used only for calculating *R*-factors(gt) *etc*. and is not relevant to the choice of reflections for refinement. *R*-factors based on *F*^2^ are statistically about twice as large as those based on *F*, and *R*- factors based on ALL data will be even larger.

### Fractional atomic coordinates and isotropic or equivalent isotropic displacement parameters (Å^2^)

**Table:**

	*x*	*y*	*z*	*U*_iso_*/*U*_eq_	Occ. (<1)
O5	1.3003 (4)	0.14269 (11)	0.28007 (10)	0.0574 (6)	
O6A	1.0846 (17)	0.2123 (3)	0.1437 (3)	0.0622 (16)	0.517 (9)
O7A	0.9012 (16)	0.0976 (4)	0.0315 (3)	0.0726 (16)	0.517 (9)
O8	1.4309 (4)	0.06126 (12)	0.40897 (9)	0.0602 (6)	
N3	0.9800 (5)	0.12260 (17)	0.11120 (12)	0.0517 (8)	
N4	1.1499 (6)	−0.09612 (17)	0.42380 (13)	0.0584 (7)	
C8	1.0438 (5)	−0.03373 (16)	0.28965 (12)	0.0398 (6)	
C9	1.1008 (5)	0.05167 (16)	0.24507 (12)	0.0390 (6)	
C10	0.9365 (5)	0.03748 (17)	0.16144 (13)	0.0416 (7)	
C11	0.7300 (6)	−0.05660 (19)	0.12321 (14)	0.0488 (8)	
C12	0.6838 (6)	−0.1393 (2)	0.16654 (14)	0.0522 (8)	
C13	0.8391 (6)	−0.12754 (18)	0.24892 (14)	0.0476 (8)	
C14	1.2174 (5)	−0.02094 (17)	0.37827 (13)	0.0442 (7)	
O7B	1.2731 (16)	0.1761 (5)	0.1186 (3)	0.0686 (17)	0.483 (9)
O6B	0.7212 (15)	0.1376 (4)	0.0676 (4)	0.0765 (19)	0.483 (9)
O1	1.1680 (4)	0.64772 (12)	0.23085 (10)	0.0576 (6)	
O2	1.6092 (5)	0.69953 (13)	0.37229 (11)	0.0717 (7)	
O3	1.3820 (6)	0.64564 (16)	0.47695 (12)	0.0965 (9)	
O4	0.7934 (4)	0.56454 (13)	0.09498 (10)	0.0650 (6)	
N1	1.4064 (5)	0.63841 (15)	0.40149 (12)	0.0521 (7)	
N2	0.5075 (6)	0.40043 (18)	0.07421 (14)	0.0632 (8)	
C1	0.8539 (5)	0.47141 (16)	0.21067 (13)	0.0412 (7)	
C2	1.0752 (5)	0.55872 (16)	0.26058 (13)	0.0409 (7)	
C3	1.1871 (5)	0.54999 (16)	0.34496 (13)	0.0418 (7)	
C4	1.0917 (6)	0.45937 (19)	0.37779 (15)	0.0492 (8)	
C5	0.8859 (6)	0.37445 (19)	0.32808 (15)	0.0543 (8)	
C6	0.7662 (6)	0.38048 (18)	0.24564 (14)	0.0482 (8)	
C7	0.7143 (6)	0.48026 (19)	0.12263 (14)	0.0475 (8)	
H4B	0.984 (7)	−0.1544 (19)	0.4054 (15)	0.0701*	
H5O	1.38739	0.13813	0.32790	0.0689*	
H12	0.533 (7)	−0.208 (2)	0.1414 (15)	0.075 (7)*	
H13	0.808 (6)	−0.1821 (18)	0.2792 (14)	0.0571*	
H11	0.632 (6)	−0.0653 (17)	0.0674 (15)	0.0586*	
H4A	1.260 (7)	−0.0911 (19)	0.4746 (17)	0.0701*	
H1O	1.07785	0.64178	0.18153	0.0692*	
H2A	0.444 (7)	0.347 (2)	0.0911 (17)	0.0758*	
H2B	0.420 (6)	0.4100 (19)	0.0199 (17)	0.0758*	
H4	1.161 (6)	0.4557 (17)	0.4324 (15)	0.0589*	
H5	0.820 (6)	0.3154 (19)	0.3482 (15)	0.0651*	
H6	0.614 (6)	0.3254 (18)	0.2137 (14)	0.0578*	

### Atomic displacement parameters (Å^2^)

**Table:**

	*U*^11^	*U*^22^	*U*^33^	*U*^12^	*U*^13^	*U*^23^
O5	0.0718 (10)	0.0450 (10)	0.0451 (9)	−0.0112 (8)	−0.0179 (8)	0.0107 (7)
O6A	0.079 (3)	0.052 (3)	0.053 (2)	0.000 (2)	−0.002 (2)	0.0178 (19)
O7A	0.075 (3)	0.097 (3)	0.038 (2)	−0.010 (2)	−0.011 (2)	0.021 (2)
O8	0.0729 (10)	0.0528 (10)	0.0431 (9)	−0.0137 (8)	−0.0177 (8)	0.0098 (8)
N3	0.0482 (12)	0.0617 (15)	0.0429 (12)	0.0011 (10)	−0.0054 (9)	0.0157 (11)
N4	0.0716 (14)	0.0557 (13)	0.0403 (11)	−0.0074 (10)	−0.0156 (10)	0.0154 (10)
C8	0.0405 (10)	0.0413 (12)	0.0345 (11)	0.0025 (9)	−0.0022 (9)	0.0042 (10)
C9	0.0362 (10)	0.0402 (12)	0.0362 (11)	0.0001 (9)	−0.0040 (8)	0.0032 (9)
C10	0.0379 (10)	0.0486 (14)	0.0373 (12)	0.0035 (9)	−0.0005 (9)	0.0108 (10)
C11	0.0467 (12)	0.0599 (16)	0.0330 (12)	−0.0004 (11)	−0.0075 (10)	0.0023 (11)
C12	0.0548 (13)	0.0498 (15)	0.0428 (14)	−0.0073 (11)	−0.0062 (10)	0.0011 (12)
C13	0.0542 (13)	0.0437 (14)	0.0406 (13)	−0.0031 (10)	−0.0013 (10)	0.0086 (10)
C14	0.0482 (12)	0.0459 (13)	0.0353 (12)	0.0034 (10)	−0.0036 (9)	0.0062 (10)
O7B	0.060 (3)	0.071 (3)	0.070 (3)	−0.016 (2)	−0.008 (2)	0.033 (2)
O6B	0.064 (3)	0.092 (3)	0.074 (4)	0.010 (2)	−0.020 (3)	0.037 (3)
O1	0.0725 (11)	0.0494 (10)	0.0490 (10)	−0.0047 (8)	−0.0044 (8)	0.0242 (8)
O2	0.0808 (12)	0.0613 (12)	0.0630 (11)	−0.0209 (9)	0.0007 (9)	0.0152 (9)
O3	0.1297 (18)	0.0999 (16)	0.0415 (11)	−0.0298 (13)	−0.0012 (11)	0.0073 (10)
O4	0.0848 (11)	0.0577 (11)	0.0489 (10)	−0.0052 (9)	−0.0095 (8)	0.0257 (8)
N1	0.0565 (11)	0.0520 (12)	0.0454 (12)	0.0023 (9)	−0.0024 (9)	0.0127 (10)
N2	0.0773 (14)	0.0598 (15)	0.0466 (12)	−0.0077 (12)	−0.0122 (10)	0.0217 (11)
C1	0.0417 (11)	0.0458 (13)	0.0385 (12)	0.0082 (9)	0.0049 (9)	0.0141 (10)
C2	0.0428 (11)	0.0416 (12)	0.0412 (12)	0.0061 (9)	0.0066 (9)	0.0160 (10)
C3	0.0416 (11)	0.0439 (13)	0.0406 (12)	0.0054 (9)	0.0041 (9)	0.0117 (10)
C4	0.0521 (12)	0.0568 (15)	0.0406 (13)	0.0065 (11)	0.0005 (10)	0.0206 (12)
C5	0.0604 (14)	0.0493 (15)	0.0530 (15)	−0.0013 (12)	−0.0022 (11)	0.0242 (12)
C6	0.0495 (13)	0.0465 (14)	0.0468 (14)	0.0007 (10)	−0.0010 (10)	0.0137 (11)
C7	0.0494 (12)	0.0520 (14)	0.0424 (13)	0.0063 (11)	0.0028 (10)	0.0158 (11)

### Geometric parameters (Å, °)

**Table:**

O5—C9	1.330 (3)	C8—C13	1.390 (3)
O6A—N3	1.203 (5)	C8—C14	1.485 (3)
O6B—N3	1.214 (6)	C8—C9	1.409 (3)
O7A—N3	1.268 (5)	C9—C10	1.400 (3)
O7B—N3	1.220 (7)	C10—C11	1.384 (3)
O8—C14	1.256 (3)	C11—C12	1.364 (3)
O5—H5O	0.8200	C12—C13	1.373 (3)
O1—C2	1.331 (3)	C11—H11	0.92 (2)
O2—N1	1.208 (3)	C12—H12	1.00 (3)
O3—N1	1.215 (3)	C13—H13	0.92 (2)
O4—C7	1.251 (3)	C1—C7	1.487 (3)
O1—H1O	0.8200	C1—C2	1.408 (3)
N3—C10	1.462 (3)	C1—C6	1.394 (3)
N4—C14	1.315 (3)	C2—C3	1.405 (3)
N4—H4B	0.91 (3)	C3—C4	1.377 (3)
N4—H4A	0.87 (3)	C4—C5	1.362 (3)
N1—C3	1.459 (3)	C5—C6	1.374 (3)
N2—C7	1.313 (3)	C4—H4	0.90 (2)
N2—H2B	0.93 (3)	C5—H5	0.89 (2)
N2—H2A	0.80 (3)	C6—H6	0.91 (2)
			
C9—O5—H5O	109.00	O8—C14—N4	120.4 (2)
C2—O1—H1O	109.00	O8—C14—C8	119.73 (19)
O6A—N3—C10	121.8 (3)	C10—C11—H11	120.5 (14)
O7A—N3—C10	116.8 (3)	C12—C11—H11	119.2 (14)
O7B—N3—C10	117.3 (3)	C11—C12—H12	122.5 (14)
O6B—N3—O7B	124.6 (4)	C13—C12—H12	118.2 (14)
O6A—N3—O7A	121.4 (4)	C8—C13—H13	117.7 (14)
O6B—N3—C10	118.1 (3)	C12—C13—H13	120.4 (14)
C14—N4—H4A	120.6 (17)	C6—C1—C7	122.08 (19)
H4A—N4—H4B	116 (2)	C2—C1—C6	119.27 (19)
C14—N4—H4B	123.0 (15)	C2—C1—C7	118.64 (19)
O2—N1—C3	119.37 (18)	O1—C2—C3	120.55 (18)
O3—N1—C3	117.86 (19)	C1—C2—C3	117.33 (19)
O2—N1—O3	122.7 (2)	O1—C2—C1	122.09 (18)
H2A—N2—H2B	120 (2)	N1—C3—C2	120.54 (18)
C7—N2—H2B	117.6 (15)	N1—C3—C4	117.60 (19)
C7—N2—H2A	122.1 (19)	C2—C3—C4	121.9 (2)
C9—C8—C13	119.43 (18)	C3—C4—C5	120.2 (2)
C13—C8—C14	122.27 (19)	C4—C5—C6	119.7 (2)
C9—C8—C14	118.26 (18)	C1—C6—C5	121.6 (2)
O5—C9—C10	120.59 (19)	O4—C7—C1	119.6 (2)
C8—C9—C10	117.21 (19)	N2—C7—C1	120.3 (2)
O5—C9—C8	122.19 (17)	O4—C7—N2	120.1 (2)
N3—C10—C11	117.63 (19)	C3—C4—H4	120.7 (15)
N3—C10—C9	120.55 (19)	C5—C4—H4	119.1 (14)
C9—C10—C11	121.8 (2)	C4—C5—H5	121.6 (15)
C10—C11—C12	120.3 (2)	C6—C5—H5	118.7 (15)
C11—C12—C13	119.3 (2)	C1—C6—H6	118.7 (15)
C8—C13—C12	122.0 (2)	C5—C6—H6	119.6 (15)
N4—C14—C8	119.9 (2)		
			
O6A—N3—C10—C9	−17.2 (5)	C9—C10—C11—C12	−0.6 (3)
O6A—N3—C10—C11	163.1 (4)	N3—C10—C11—C12	179.1 (2)
O7A—N3—C10—C9	163.0 (4)	C10—C11—C12—C13	1.2 (4)
O7A—N3—C10—C11	−16.7 (4)	C11—C12—C13—C8	−0.2 (4)
O2—N1—C3—C2	−30.7 (3)	C6—C1—C2—O1	−179.93 (18)
O2—N1—C3—C4	150.2 (2)	C6—C1—C2—C3	−2.0 (3)
O3—N1—C3—C2	151.1 (2)	C7—C1—C2—O1	−1.3 (3)
O3—N1—C3—C4	−28.0 (3)	C7—C1—C2—C3	176.59 (19)
C13—C8—C9—C10	1.7 (3)	C2—C1—C6—C5	1.0 (3)
C14—C8—C9—O5	−0.7 (3)	C7—C1—C6—C5	−177.5 (2)
C13—C8—C9—O5	−178.57 (19)	C2—C1—C7—O4	−0.5 (3)
C9—C8—C14—N4	175.1 (2)	C2—C1—C7—N2	179.9 (2)
C13—C8—C14—O8	172.1 (2)	C6—C1—C7—O4	178.1 (2)
C13—C8—C14—N4	−7.1 (3)	C6—C1—C7—N2	−1.5 (3)
C14—C8—C13—C12	−179.0 (2)	O1—C2—C3—N1	0.1 (3)
C14—C8—C9—C10	179.55 (18)	O1—C2—C3—C4	179.2 (2)
C9—C8—C13—C12	−1.3 (3)	C1—C2—C3—N1	−177.82 (18)
C9—C8—C14—O8	−5.6 (3)	C1—C2—C3—C4	1.3 (3)
O5—C9—C10—N3	−0.3 (3)	N1—C3—C4—C5	179.7 (2)
O5—C9—C10—C11	179.5 (2)	C2—C3—C4—C5	0.6 (3)
C8—C9—C10—N3	179.46 (18)	C3—C4—C5—C6	−1.7 (4)
C8—C9—C10—C11	−0.8 (3)	C4—C5—C6—C1	0.9 (4)

### Hydrogen-bond geometry (Å, °)

**Table:**

*D*—H···*A*	*D*—H	H···*A*	*D*···*A*	*D*—H···*A*
O1—H1O···O4	0.82	1.77	2.500 (2)	148
N2—H2A···O6A^i^	0.80 (3)	2.38 (3)	3.142 (5)	161 (3)
N2—H2B···O4^ii^	0.93 (3)	2.03 (3)	2.966 (3)	177 (2)
N4—H4A···O8^iii^	0.87 (3)	2.07 (3)	2.929 (3)	172 (2)
N4—H4B···O2^iv^	0.91 (3)	2.18 (3)	3.084 (3)	174 (2)
O5—H5O···O8	0.82	1.76	2.496 (2)	148
C6—H6···O6A^i^	0.91 (2)	2.40 (2)	3.285 (6)	163 (2)
C11—H11···O7A^v^	0.92 (2)	2.41 (2)	3.221 (6)	147 (2)
C11—H11···O7A^vi^	0.92 (2)	2.55 (2)	2.997 (6)	110.5 (17)
C13—H13···O2^iv^	0.92 (2)	2.39 (2)	3.289 (3)	165 (2)

Symmetry codes: (i) *x*−1, *y*, *z*; (ii) −*x*+1, −*y*+1, −*z*; (iii) −*x*+3, −*y*, −*z*+1; (iv) *x*−1, *y*−1, *z*; (v) −*x*+1, −*y*, −*z*; (vi) −*x*+2, −*y*, −*z*.
